# Intraoperative Seizure Under General Anesthesia Not Detected by EEG: A Case Report

**DOI:** 10.7759/cureus.42765

**Published:** 2023-07-31

**Authors:** Sumanya Kumar, Alexander J Rodriguez, Mark A Burbridge

**Affiliations:** 1 Anesthesiology, University of Connecticut School of Medicine, Farmington, USA; 2 Anesthesiology, Stanford University Medical Center, Stanford, USA

**Keywords:** propofol, anesthesia, intraoperative, eeg, seizure

## Abstract

Intraoperative seizures under general anesthesia are infrequent. However, seizure activity under general anesthesia confirmed by contemporaneous EEG has been reported. We describe the case of a 39-year-old female undergoing right frontal brain tumor resection who experienced an intraoperative seizure. Intraoperative neuromonitoring was utilized and included four channels of EEG, somatosensory evoked potentials (SSEP), and transcranial motor evoked potentials (MEP). During this operation, characteristic motor manifestations of a seizure occurred. However, the EEG did not demonstrate seizure activity due to limitations in EEG lead placement. Post-operatively in the ICU, motor manifestations of seizure activity continued, and subsequent EEG recordings demonstrated classic seizure activity. Due to the previous hemicraniectomy, corkscrew EEG electrodes were not placed over the right skull defect, thereby failing to detect the intraoperative seizure. Anesthesiologists should be aware that limitations with EEG electrode placement can fail to detect intraoperative seizures, and treatment to extinguish the seizure should proceed in an emergent fashion.

## Introduction

Intraoperative seizures have been confirmed with contemporaneous EEG under general anesthesia [1] using many different anesthetic regimens, including volatile anesthetics [2,3] and propofol [4-6]. Here, we present the case of a focal motor seizure in a patient undergoing a right frontal meningioma resection who had no history of seizures. Interestingly, this motor seizure was not registered despite four channels of contemporaneous EEG due to a large previous hemicraniectomy which limited EEG lead placement over the operative area. Still, because of the high risk of leaving a seizure untreated in this case, it was extinguished with intravenous administration of propofol and levetiracetam. A post-operative EEG showed classic seizure activity.

Typically, the gold standard for seizure detection is EEG, but the absence of EEG electrodes over the previous hemicraniectomy defect necessitated utilizing expert clinical examination to diagnose the seizure for prompt treatment. Anesthesiologists should be aware that intraoperative seizures may occur in patients under general anesthesia and that limitations in EEG lead placement may require seizure treatment even in the absence of EEG findings.

## Case presentation

The patient was a 39-year-old female presenting for the surgical resection of a 1 cm by 1 cm right frontal meningioma and the replacement of a bone flap. Her past medical history included a three-month prior right frontal craniotomy to resect an anterior falcine meningioma. This was complicated by subsequent herniation, subarachnoid hemorrhage (SAH), perilesional infarction, and right lateral ventricle intraventricular hemorrhage. Due to an acute rise in intracranial pressure and neurological deterioration, she underwent an emergent hemicraniectomy later that day. One month later, she then developed hydrocephalus requiring a ventriculoperitoneal shunt. She had no other documented medical history, including no history of seizures, her pre-operative laboratory values were within normal limits, and she was on no medications. Her physical examination was normal aside from a mild left facial droop and mild left upper and left lower extremity (LLE) weakness.

Pre-operatively, the patient received 2 mg of midazolam IV for anxiolysis. The patient was transported to the operating room where standard monitors were applied. Anesthesia was induced with fentanyl 150 mcg IV, propofol 100 mg IV, and rocuronium 40 mg IV, followed by endotracheal intubation and arterial line placement. Neurophysiologic monitors were placed, including four EEG channels, somatosensory evoked potentials (SSEP), and transcranial motor evoked potentials (MEP) bilaterally in the upper and lower extremities. Importantly, no right frontal EEG leads could be placed given the prior hemicraniectomy. The patient was then maintained on propofol 50 mcg/kg/min, 0.5 MAC sevoflurane, and remifentanil 0.05-0.2 mcg/kg/min. Dexamethasone 8 mg and cefazolin 2 g were administered before the surgical incision. The anesthetic regimen at our institution is at the discretion of the anesthesiology team, and a total intravenous regimen is not required. The intraoperative blood pressure goal included a mean arterial pressure range of 70-90 mmHg.

The patient was positioned supine and rotated 180 degrees in the usual institutional fashion. The patient’s head was carefully placed in the three-point fixation frame, with special attention to avoid the previous craniectomy. The intraoperative neuromonitoring team then established satisfactory and reproducible neuromonitoring potentials, with the limitation that the EEG electrodes were not able to be placed over the right-sided previous craniectomy location. Approximately 17 minutes after the incision, but before the opening of the dura, the anesthesiologist observed rhythmic shaking movements of the LLE. A seizure was considered the most likely diagnosis after conferring with the surgical and neurology teams, and propofol 50 mg IV was bolused with a prompt resolution of the movements. Levetiracetam 1 g was then administered for seizure prophylaxis. The rhythmic movements of the LLE occurred again approximately 30 minutes later and just before emergence and again were aborted with propofol 50 mg IV bolus. Following the second event, the propofol infusion was increased to 100 mcg/kg/min. No epileptiform activity was evident on EEG up to this point in the case. The patient was extubated uneventfully in the operating room in order to obtain a swift neurologic examination. No other TcMEp or SSEP changes were noted. The surgery was completed with a complete resection of the brain tumor.

**Figure 1:**
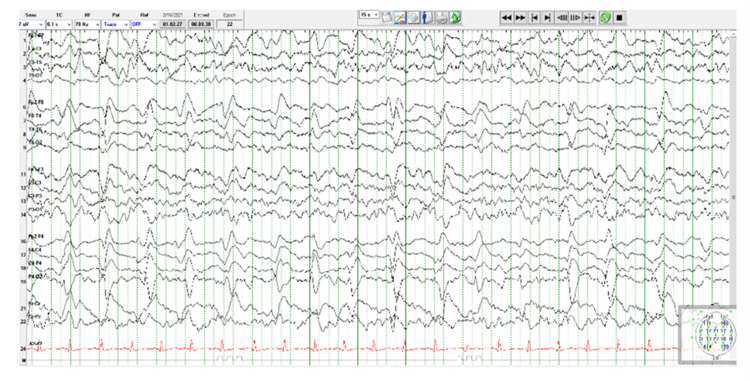
EEG within minutes of arrival to post-operative ICU

**Figure 2:**
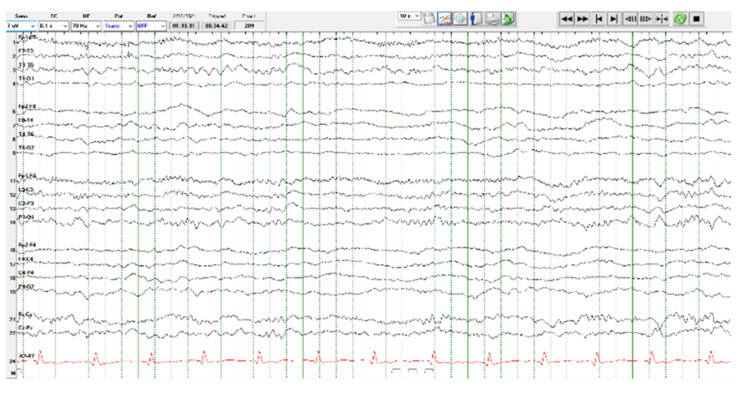
EEG post-lorazepam, 16 minutes after EEG 1

The EEG obtained post-operatively in the ICU (Figure 1) with no additional sedation administered demonstrated mild-moderate slowing, continuous right central-parietal slowing, and frequent epileptiform discharges. Two mg IV lorazepam was administered to extinguish seizure activity (Figure 2). Regularly scheduled levetiracetam was initiated for prophylaxis for subsequent episodes of seizure activity. Both EEGs were performed with a digital EEG machine with video, 24 channels, with standard international 10-20 system electrode placements, eye movement leads, and an EKG lead. The patient showed increased left upper extremity weakness post-operatively, which gradually resolved over the next two days in the ICU. On post-operative day three, she was transferred out of the ICU to the neurosurgery ward and then was transferred home on post-operative day four at her baseline neurological status.

## Discussion

Intraoperative seizures are infrequent under general anesthesia [7]. When they do occur, they have been recorded using direct clinical observation, electromyography [8], and EEG [1]. EEG is, however, considered the gold standard for detecting and diagnosing seizures. The detection of seizures through EEG is highly dependent on proper lead placement to monitor the desired anatomical territories accurately [4]. In the presented case, however, the placement of EEG electrodes over the right frontal hemisphere was not possible because of the prior craniectomy over that anatomical location. The intraoperative EEG was recorded from four channels. There were no changes noted on the EEG at any time during the surgical procedure to clearly indicate a focal ischemic process, epileptiform discharges, or seizures. The post-operative EEG, however, demonstrated classic seizure activity with motor manifestations making the diagnosis of intraoperative seizure very likely.

The differential diagnosis for patient movement under general anesthesia can include light anesthesia, myoclonus from propofol administration, shivering, and seizures. Light anesthesia was considered unlikely because the EEG indicated that the patient was adequately anesthetized according to the interpretation by the attending neurologist. In addition, the mean core body temperature during the case was 36.4 C which made the possibility of shivering unlikely. Therefore, the two leading differential diagnoses were an intraoperative seizure and myoclonus from propofol. In the described case, the patient had several risk factors for seizures, including recent craniotomy, SAH, and cerebral infarction, which, when considered with the patient's clinical picture, make the likelihood of a seizure high, although she had never actually experienced a seizure prior to surgery. No other risk factors for seizure activity were present, as the patient's baseline laboratory results were unremarkable, including electrolyte, complete blood count, urea, creatinine, and glucose, making metabolic abnormalities during the operation unlikely. The patient had no known source of infection. A toxicology screening was not performed as a potential etiology for seizure activity, but no other withdrawal symptoms were noted intraoperatively or post-operatively and the patient reported no alcohol or illicit drug use in the pre-operative interview.

Myoclonus from propofol administration can present with motor manifestations that are difficult to distinguish from seizure activity. This myoclonic activity from propofol, however, would not display any significant EEG findings, however. Multiple case reports of seizure-like myoclonic activity after propofol administration have been reported. Hickey et al. discussed a 78-year-old male undergoing left hip arthroplasty with no significant past medical history and laboratory studies within normal limits. Thirty seconds after initiation of propofol induction, he began having generalized movements of the upper and lower body that ceased after sedation was stopped and intravenous diazepam was administered. An EEG was then applied and showed no evidence of seizure activity, but this was after the movements had stopped making the diagnosis of seizure unclear [6].

Yi et al. [9] published the case of a 23-year-old male with no significant past medical history, illicit drug or alcohol use, or laboratory abnormalities undergoing surgery for the removal of a pilonidal cyst. After induction of anesthesia, he displayed rhythmic tonic-clonic movements that were so severe that the case was canceled because of a high suspicion of an intraoperative seizure. He suffered no seizure activity post-operatively and was fully investigated from a neurological point of view with no explanation for the shaking movements. On the basis of this uncertainty, the next case was initiated with EEG applied. After induction of anesthesia with propofol, the patient had a similar recurrent episode of generalized tonic-clonic movements that were extinguished with propofol boluses. Intraoperative EEG did not show evidence of seizure activity, making myoclonus from propofol administration the most likely explanation.

However, seizure activity under general anesthesia has been recorded during neurosurgical procedures. A published care report described a 34-year-old man who underwent a craniotomy for the resection of an arteriovenous malformation [1]. While the craniotomy was being performed, the patient suffered a tonic-clonic seizure that was captured by intraoperative EEG. The seizure was treated with a propofol bolus. The patient had no previous history of seizures, and no cause for the seizure was identified. The seizure occurred, similarly to the case presented, during the craniotomy and before the dura had been opened, making surgical factors unlikely to be contributory to the seizure. Interestingly, this patient was run on the same intraoperative anesthetic regimen as in the case report presented. This was the first case of an intraoperative seizure occurring under general anesthesia captured by intraoperative EEG during a neurosurgical procedure.

A retrospective report [10] investigated 400 consecutive neurosurgical patients who underwent craniotomy for a variety of procedures under general anesthesia with total intravenous anesthesia with a propofol infusion. None of the patients had any risk factors for seizures and did not have intracranial pathology that placed them at elevated risk of seizures. When the EEG records for these cases were examined, two of the 400 patients (0.5%) showed evidence of seizure activity.

The clinical presentation of an intraoperative seizure and myoclonus from propofol is very similar and virtually indistinguishable without accurate and contemporaneous EEG. Although there was no intraoperative EEG indication of a seizure in our case, treating the seizure activity emergently based on the characteristic motor manifestations was decided upon, given the limitations of EEG electrode placement and the urgent need to extinguish the seizure. The presence of seizure activity captured immediately post-operatively makes it likely that the patient did in fact have experienced an intraoperative seizure.

There are currently no published guidelines regarding the treatment of intraoperative seizures. In this case, propofol bolus successfully extinguished seizure activity. Anesthesiologists should be aware of the potential for intraoperative seizure, especially considering patient risk factors, utilization of certain induction agents, and the limitations of EEG placement for intraoperative seizure diagnosis.

## Conclusions

We report for the first time the case of an intraoperative motor seizure that was not registered on the contemporaneous four channels of EEG under general anesthesia. The reason for the failure to detect this seizure with EEG was due to the need to place the EEG electrodes away from the prior surgical site of a hemicraniectomy, thereby leaving the underlying brain, where the seizure originated, unmonitored. Vigilant intraoperative monitoring of the patient by the anesthesiology and neurology teams was instrumental in detecting the motor manifestations of the seizure. Due to a high degree of clinical suspicion based on the motor manifestations from the seizure, and the acknowledgment that the EEG lead placement was not optimal to detect a seizure over the previous hemicraniectomy site, the seizure was promptly extinguished with a propofol bolus. The primary lesson of this case report is that anesthesiologists should be aware of the limitations of intraoperative EEG in detecting seizures due to lead placement and should be prepared to treat seizures expeditiously. While EEG is the gold standard for the diagnosis of seizures intraoperatively, the limitations of EEG lead placement as illustrated in this case report can necessitate the use of clinical examination to diagnose and treat seizure activity.
